# Isobutanol production from cellobionic acid in *Escherichia coli*

**DOI:** 10.1186/s12934-015-0232-6

**Published:** 2015-04-15

**Authors:** Shuchi H Desai, Christine A Rabinovitch-Deere, Zhiliang Fan, Shota Atsumi

**Affiliations:** Department of Chemistry, University of California, Davis, One Shields Ave, Davis, CA 95616 USA; Microbiology Graduate Group, University of California, Davis, One Shields Ave, Davis, CA 95616 USA; Department of Biological and Agricultural Engineering, University of California, Davis, One Shields Ave, Davis, CA 95616 USA

**Keywords:** Cellobionic acid, Metabolic engineering, Isobutanol

## Abstract

**Background:**

Liquid fuels needed for the global transportation industry can be produced from sugars derived from plant-based lignocellulosics. Lignocellulosics contain a range of sugars, only some of which (such as cellulose) have been shown to be utilizable by microorganisms capable of producing biofuels. Cellobionic acid makes up a small but significant portion of lignocellulosic degradation products, and had not previously been investigated as an utilizable substrate. However, aldonic acids such as cellobionic acid are the primary products of a promising new group of lignocellulosic-degrading enzymes, which makes this compound group worthy of study. Cellobionic acid doesn’t inhibit cellulose degradation enzymes and so its inclusion would increase lignocellulosic degradation efficiency. Also, its use would increase overall product yield from lignocellulose substrate. For these reasons, cellobionic acid has gained increased attention for cellulosic biofuel production.

**Results:**

This study describes the discovery that *Escherichia coli* are naturally able to utilize cellobionic acid as a sole carbon source with efficiency comparable to that of glucose and the construction of an *E. coli* strain able to produce the drop-in biofuel candidate isobutanol from cellobionic acid. The gene primarily responsible for growth of *E. coli* on cellobionic acid is *ascB*, a gene previously thought to be cryptic (expressed only after incurring specific mutations in nearby regulatory genes). In addition to AscB, the *ascB* knockout strain can be complemented by the cellobionic acid phosphorylase from the fungus *Neurospora crassa*. An *E. coli* strain engineered to express the isobutanol production pathway was successfully able to convert cellobionic acid into isobutanol. Furthermore*,* to demonstrate potential application of this strain in a sequential two-step bioprocessing system, *E. coli* was grown on hydrolysate (that was degraded by a fungus) and was successfully able to produce isobutanol.

**Conclusions:**

These results demonstrate that cellobionic acid is a viable carbon source for biofuel production. This work suggests that with further optimization, a bacteria-fungus co-culture could be used in decreased-cost biomass-based biofuel production systems.

## Background

Liquid fuel is an essential component of today’s modern world that allows global economies, travel, and daily tasks to occur with ease. There is increasing momentum towards the search and development of alternative liquid fuel sources traditionally made from petroleum due to concerns about the finite petroleum supply, national security, and ecological impact [1].

Biofuels, liquid fuels made by conversion of biomass, are a renewable alternative for petroleum-based transportation fuels. Lignocellulosics, the component of plant biomass typically discarded as waste, can be used as substrate for biofuel production and could help recycle carbon dioxide emitted from fuel combustion, potentially decreasing greenhouse gases and making biofuels carbon neutral [2,3]. The use of non-food crop biomass would circumvent disturbing the food supply, crop prices, and arable land space for food crops [4-6]. Biochemical conversion of lignocellulosic biomass to fuels and chemicals begins with enzymatic hydrolysis by cellulase enzymes and the production of sugars as the substrate for subsequent microbial fermentation. [7]. The high cost of cellulase enzymes remains one of the bottlenecks for a low cost processing technology [8]. Microorganisms used in consolidated bioprocessing systems, in which a single microbe converts lignocellulosics to simple sugars and then to biofuels, can mitigate that bottleneck [9,10], reduce cost and increase efficiency [7].

Recent studies focusing on lignocellulosic degradation have identified lytic polysaccharide monooxygenase (LPMO) enzymes that can significantly increase the activity of the cellulase cocktail and accelerate cellulose degradation [11,12]. Aldonic acids (sugar acids with a carboxylic acid group at the terminal carbon), including cellobionic acid (CBA), are the main product of the LPMO-assisted oxidative hydrolysis of cellulose polymers. Growing research interest in the benefits of LPMOs in lignocellulosic degradation also arouses interest in microbial utilization of the resulting aldonic sugars. Aldonic sugars can also be formed spontaneously in fermentation settings and was considered lost sugar if they were not utilized as substrate for microbial fermentation [13]. Therefore, it is of great interest to investigate the microbial utilization of these otherwise lost carbon sources to improve process economics.

CBA is of specific interest because it is similar in structure to cellobiose, a prevalent product of lignocellulosic degradation. CBA also has less feedback inhibition on cellulases as compared to cellobiose [14], which may allow for less enzyme required in the enzymatic step of lignocellulosic degradation, which in turn would decrease processing costs.

Aldonic sugars have also been proposed as an alternative substrate to sugars for fuel and chemical production. Fan et al. proposed a novel route of biofuels and chemicals production, in which cellulase production and enzymatic hydrolysis is combined into one step using a genetically engineered fungus strain [9,15]. Aldonic sugars were produced directly from cellulose by the engineered fungus strain without exogenous cellulase production. It was demonstrated that both glucose and gluconate, the products from CBA hydrolysis, can be used as the carbon source for biofuel production by a recombinant *E. coli* strain [15]. However, the possibility of directly using CBA as the carbon source for microbial fermentation was not yet investigated.

**Figure 1:**
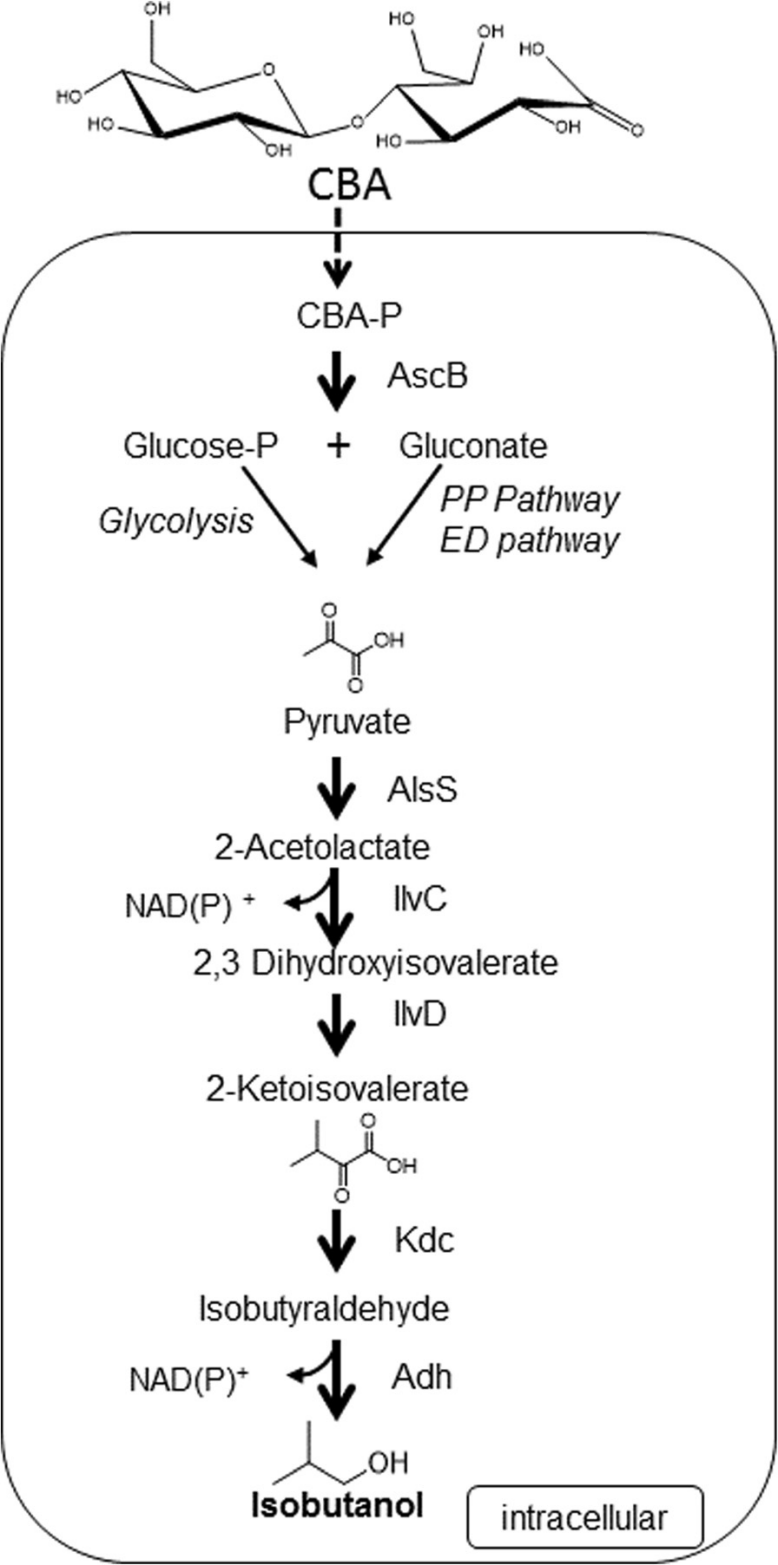
**Pathway for biological conversion of cellobionic acid to isobutanol.** Cellobionic acid is naturally imported into the cell and is most likely broken down into glucose 6-phosphate and gluconate. These sugars are converted by the modified valine biosynthesis pathway to produce isobutanol. PP; pentose phosphate, ED; Entner Doudoroff, CBA; cellobionic acid, CBA-P; phosphorylated cellobionic acid.

To date, very few enzymes have been identified with specific activity on aldonic acids, including a recently characterized phosphorylase from the fungus *Neurospora crassa* with activity on CBA [16]. Thus, in this study, we explored metabolic engineering options to utilize the aldonic acid CBA for target chemical production in order to find ways to curb carbon loss during fermentation. Specifically, this study aims to build upon the work of Hildebrand et al. [17] in which CBA is produced with the help of *N. crassa*. The CBA produced from this system can then be fermented in a separate vessel by another microorganism to produce a target biochemical. To complete the latter part of this platform scheme, the model organism *E. coli* was chosen due to its well-studied genetic systems, ease of genetic tractability, and fast growth rate. The biofuel candidate isobutanol was chosen as the target chemical to be produced because it is chemically similar to gasoline, and so this drop-in biofuel candidate can be stored, transported, and utilized in the same engines and other infrastructure as used for gasoline [18-20]. *E. coli* has previously been demonstrated to produce isobutanol from glucose through intermediates of the valine biosynthesis pathway [19,21] (Figure 1). The engineered pathway converted the natural L-valine precursor 2-ketoisovalerate to isobutyraldehyde by a ketoacid decarboxylase (Kdc), and then to isobutanol via an aldehyde reductase/alcohol dehydrogenase (Adh).

This study reports the investigation of the previously unknown ability of *E. coli* to metabolize the aldonic acid CBA, the identification of the primary enzyme responsible for this activity in *E. coli*, and the demonstration of isobutanol production from CBA (Figure 1). Furthermore, successful isobutanol production was achieved from crude hydrolysate made from cellulose degraded to CBA by a recombinant fungus *N. crassa*. This demonstrates that CBA is a viable substrate for biofuel and other target chemical production.

## Results

### Identifying genes responsible for natural CBA metabolism

**Figure 2:**
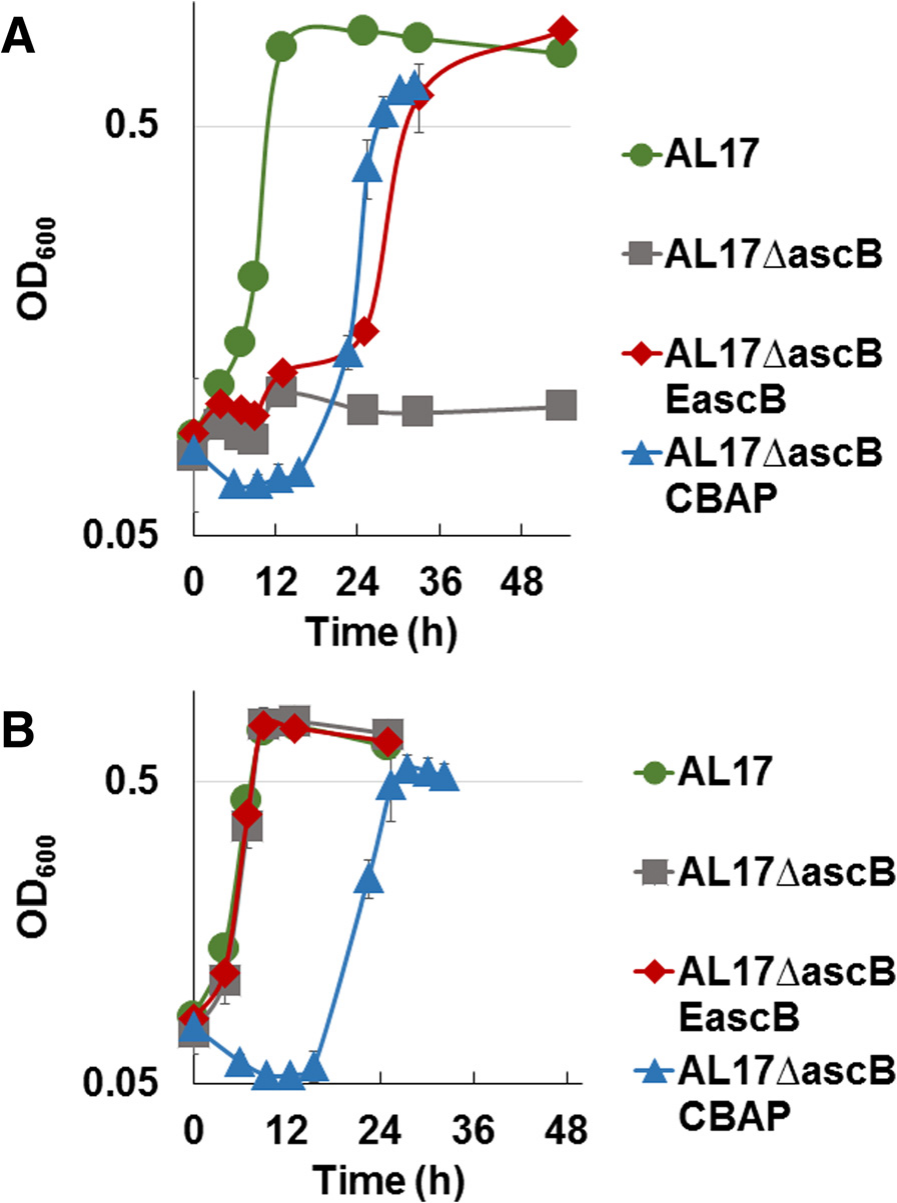
**Identification of the gene responsible for natural cellobionic acid metabolism in** ***E. coli.*** Growth of strains with CBA (**A**) or glucose (**B**). AL17 (isobutanol host strain, Table 1) (circle), AL17 with *ascB* deleted (square), AL17∆*ascB* complemented with *ascB* expression from a plasmid (EascB) (diamond), AL17∆*ascB* expressing cellobionic acid phosphorylase (CBAP) (triangle). Where n = 3 biological replicates, and error bars represent standard deviation.

Initially, a cellobionic acid phosphorylase (CBAP) from *N. crassa* [16] was expressed in *E. coli* to achieve isobutanol production directly from CBA. Surprisingly, the negative control strain (without CBAP) grew similarly in CBA as it did in glucose (Figure 2). This indicated that *E. coli* naturally imports and metabolizes CBA. To understand if other *E. coli* strains could metabolize CBA, XL-1 Blue, BL21(DE3), and MG1655 were tested for their ability to grow on CBA. These strains were also able to grow on CBA (Figure 2). Intrigued by this finding, the enzyme(s) responsible for CBA metabolism were sought. Enzymes were chosen for inactivation based on previous studies of their activity towards substrate similar in structure or size to CBA. Five genes encoding the enzymes most likely to cleave CBA, due to their previously known activity towards other disaccharides such as cellobiose, were chosen to be deleted from the *E. coli* isobutanol host strain (AL17, Table 1) genome: *ascB*, *treB*, *chbF*, *bglA*, and *bglB*. Of note is the cryptic nature of *ascB* previously described, in which *ascB* expression occurs only in conjunction with specific mutations in the *asc* operon [22]. The cryptic expression of *ascB* allowed the growth of *E. coli* on cellobiose, salicin and arbutin. Based on sequence similarity to other phosopho-beta-glucosidases, the mechanism of AscB catalysis is hypothesized to be hydrolysis [22], but this has not been experimentally verified. Deletion of *ascB* resulted in the loss of *E.coli* growth on CBA (Figure 2A), and *ascB* complementation restored the strain’s ability to grow on CBA (Figure 2A).

**Table 1:** **Strains and plasmids used in this study**

	**Relevant genotype**	**Reference**
**Strain**		
AL17	Same as JCL260	[19]
AL1963	AL17 with *ascB*::Kan^r^	This Study
MG1655	F^−^ lambda^−^ *ilvG*- *rfb-50 rph-1*	ATCC
XL-1Blue	*recA1 endA1 gyrA96 thi-1 hsdR17 supE44 relA1 lac* [F’ *proAB lacI* ^*q*^ *Z∆M15* Tn*10* (Tet^r^)]	Agilent Technologies
BL21(DE3)	F^-^ *ompT hsdSB*(*rB* ^*−*^ *, mB* ^*−*^) *gal dcm* (DE3)	NEB
**Plasmids**		
pSA69	p15A ori; Kan^R^; *P*_L_lacO_1_:*alsS-ilvCD*	[19]
pAL377	ColE1 ori; Amp^R^; *P*_L_lacO_1_	[29]
pAL450	pSC101* ori; Cm^R^; *P*_L_tetO_1_:*osmY-bglC*	[29]
pAL451	p15A ori; Cm^R^; *P*_L_tetO_1_:*osmY-bglC*	[29]
pAL590	p15A ori; Kan^R^ *P*_L_lacO_1_:*alsS-ilvCD P*_L_tetO_1_:*osmY-bglC*	This Study
pAL536	p15A ori; Cm^R^; *P*_L_tetO_1_	[29]
pAL603	ColE1 ori; Amp^R^; *P*_L_lacO_1_:*kivd-adhA, P*_L_lacO_1_:*alsS-ilvCD*	[29]
pAL856	p15A ori; Cm^R^; *P*_L_tetO_1_ :*osmY-bglC*	This Study
pAL869	p15A ori; Cm^R^; *P*_L_tetO_1_ :*osmY-CBAP*	This Study
pAL952	ColE1 ori; Amp^R^; *P*_T7_: His6x-*ascB*	This Study
pAL959	pBBR32 ori; Amp^R^; *P*_L_lacO_1_: *ascB*	This Study
pAL982	pSC101* ori; Amp^R^; *P*_L_lacO_1_:*ascB*	This Study

* The origin of replication is described in [37].

We attempted to assay the enzymatic activity of AscB on CBA in two ways, with purified enzyme and with cell lysate, but neither was successful. His-tagged AscB could not be purified since the expression was not detectable on an SDS polyacrylamide gel. *In vitro* CBA catalysis with purified enzyme may not have detectable enzyme activity anyway because AscB is a phospho-beta-glucosidase [22] and so requires phosphorylated substrate. Cell lysate would likely provide the necessary substrate (with CBA phosphorylated upon import), and so was used as a second way to attempt to assay AscB activity. However, cell lysate from the AscB complemented strain was unable to provide adequate enzyme levels, as it also failed to show a band attributable to AscB on an SDS polyacrylamide gel. Difficulty in expressing AscB efficiently in *E. coli* agrees with the previously observed phenotype of slow growth with overexpression of *ascB* [23]. The *ascB* gene was cloned onto a low copy plasmid to reduce expression of *ascB*, but the strain harboring the low copy plasmid still showed similar growth with that of the strain harboring the *ascB* high copy plasmid. This growth defect is inferred to be a result of AscB toxicity to *E. coli* [23].

As an alternate way to understand how AscB may be metabolizing CBA in *E. coli*, the *CBAP* from *N. crassa* was expressed in the *E. coli ascB* knock out strain. Extracellular expression of this gene restored the capacity of *E. coli* to grow on CBA as the sole carbon source (Figure 2A). Successful complementation by CBAP and sequence similarity to other phosopho-beta-glucosidases suggest that AscB may cleave phosphorylated CBA into sugar monomers. Further experimental analysis will elucidate the validity of this hypothesis.

### Isobutanol production from CBA

**Figure 3:**
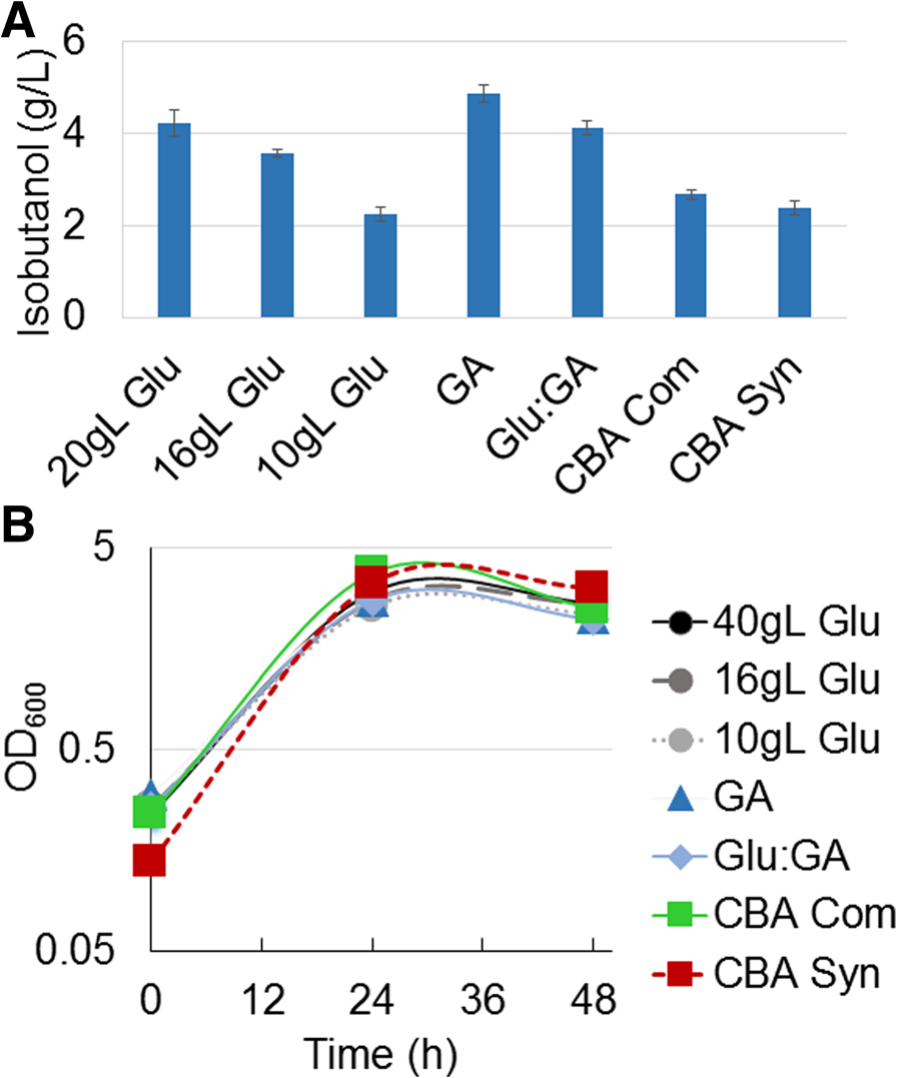
**Isobutanol Production from CBA. A.** Isobutanol titers from strain AL17(pAL603) after 24 hours of production in M9 Production Media. 20gL Glu; 20 g/L glucose, 16gL Glu; 16 g/L glucose, 10gL Glu; 10 g/L glucose, GA; 20 g/L gluconate, Glu:GA; 10 g/L glucose and 10 g/L gluconate, CBA com; 10.4 g/L commercial CBA, CBA Syn; 16.9 g/L biologically synthesized CBA. **B.** Growth of isobutanol host strain AL17(pAL603) during production period. Where n = 3 biological replicates, and error bars represent standard deviation.

Upon identifying the genes responsible for CBA metabolism, the capacity for isobutanol production from this carbon source was tested (Figure 3). CBA was hypothesized to be phosphorylated during transport and cleaved by AscB to produce glucose 6-phosphate (G6P) and gluconate [22]. In order to mimic the immediate products of CBA degradation, media with a 1:1 ratio of glucose and gluconate was tested as substrate for isobutanol production (Figure 3). Isobutanol titers from the mixed media were similar to the glucose-grown control strain. From 20 g/L gluconate, 4.9 g/L isobutanol was produced in 24 hours, achieving 70% of the theoretical maximum and a productivity of 0.20 g/L/h. From 10 g/L glucose and 10 g/L gluconate, 4.1 g/L isobutanol was produced in 24 hours, yielding 54% of the theoretical maximum and productivity of 0.17 g/L/h. From 20 g/L glucose, 4.2 g/L isobutanol was produced in 24 hours, attaining 51% of the theoretical maximum and a productivity of 0.23 g/L/h. After 24 hours of production, all the carbon was consumed (in all cultures) and none remained in the media. The similarity in isobutanol titers achieved from glucose, gluconate, and a mixture of glucose and gluconate suggest that CBA may be metabolized in the hypothesized manner. With confirmation that CBA degradation products produce isobutanol titers similar to the glucose-grown control strain, isobutanol production from CBA was then tested.

Due to the limited availability and high cost of commercial CBA, biologically synthesized CBA was used for most of the experiments herein (see Materials and Methods). Additionally, this study aims to produce isobutanol in a system applicable to industrial settings, and utilize not simple sugars such as glucose, but CBA produced from cellulose degraded by another organism [15,17]. Here we compared isobutanol titers from CBA that was from two sources: Chemical (commercial) or biological synthesis (Figure 3). Hereafter these CBA will be called commercial or synthesized, respectively. To ensure that the synthesized CBA did not contain any inhibitors that would hinder isobutanol production, a comparison of isobutanol production from both synthesized and commercial CBA was performed. Since purity analysis of commercial CBA was not available, MS analysis and HPLC measurement were performed. The commercial CBA was found to be ~56% pure and 10.4 g/ L was the actual starting concentration instead of the expected 20 g/L. From 10.4 g/L of commercial CBA, 2.7 g/L isobutanol was produced within 24 hours, yielding a productivity of 0.11 g/L/h and 65% of the theoretical maximum (Figure 3A). In comparison, from 10 g/L glucose 2.3 g/L isobutanol was produced, representing 56% of the theoretical maximum. A target concentration of 20 g/L synthesized CBA was measured by HPLC to actually be 16.9 g/L. From 16.9 g/L synthesized CBA, 2.4 g/L isobutanol was produced in 24 hours, achieving 36% of the theoretical maximum with a productivity 0.10 g/L/h. In comparison, from 16 g/L glucose 3.6 g/L isobutanol was produced, representing 55% the theoretical maximum and a productivity of 0.15 g/L/h (Figure 3A).

**Figure 4:**
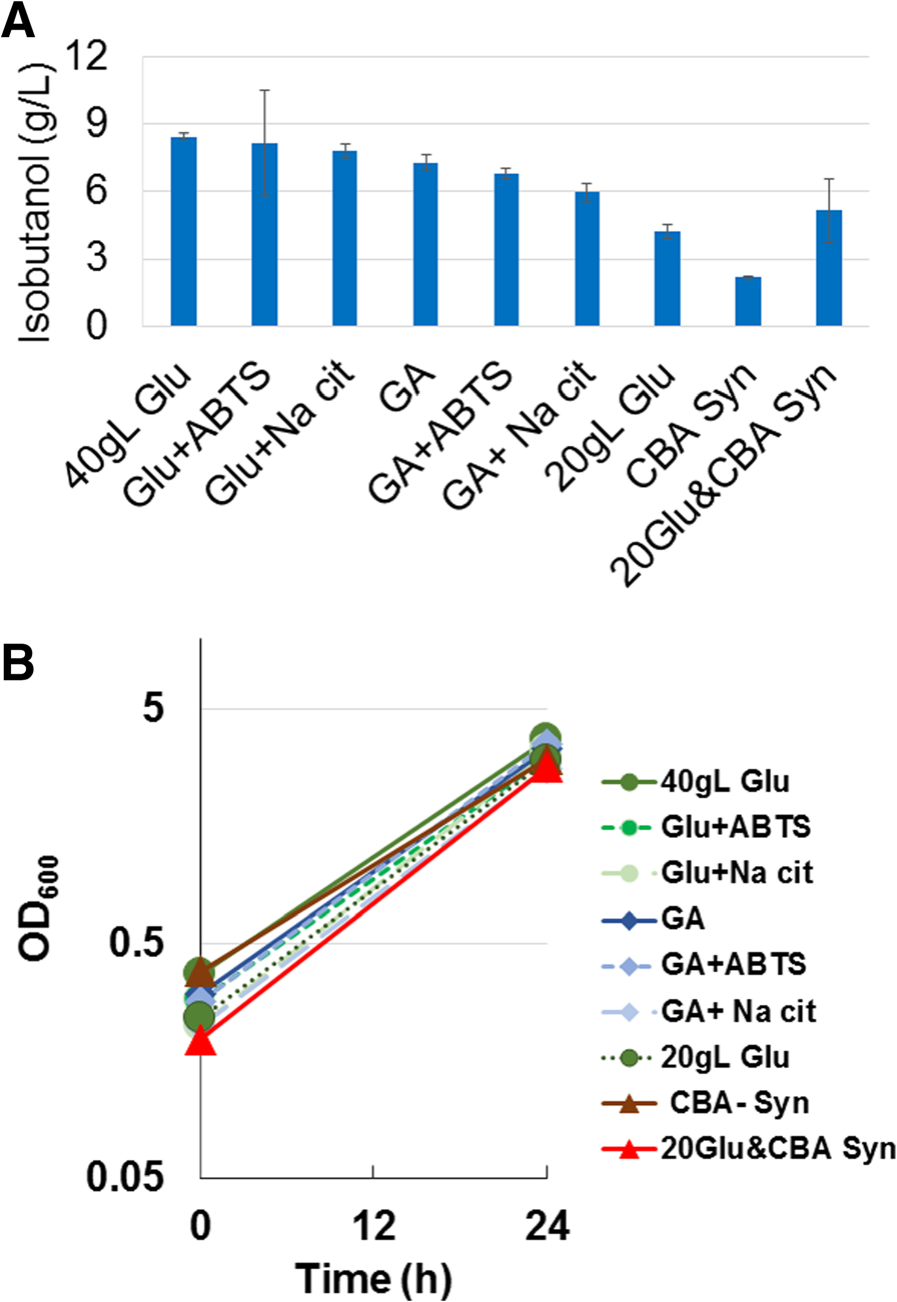
**Synthesized CBA does not have inhibitors for isobutanol production. A.** Isobutanol production from AL17(pAL603) using 20 g/L glucose (20gL Glu), 40 g/L glucose (40gL Glu), gluconate (GA) or synthesized CBA (CBA Syn). Glucose and gluconate were tested as growth substrates with or without one of the individual chemical components used in CBA synthesis (ABTS; 0.1 mM ABTS or Na Cit; 30 mM Na citrate). 20Glu&CBA Syn; 20 g/L glucose plus 40 g/L CBA Syn. **B.** Growth of AL17(pAL603) in all tested conditions. Where n = 3 biological replicates except for 20GLu& CBA Syn, n = 4, and error bars represent standard deviation.

From the above data, it appears that under the tested conditions, synthesized CBA is not converted into isobutanol as well as glucose. It was possible that components used to synthesize CBA inhibited efficient isobutanol production, and so isobutanol production was tested with each chemical separately added to production media containing either glucose or gluconate, mimicking the amount that would be in CBA production media (Figure 4). Production media with glucose or gluconate plus either ABTS or sodium citrate had similar isobutanol titers to media without these additional chemicals (Figure 4). This suggests that neither of these chemicals inhibit the consumption of glucose or gluconate, or the production of isobutanol. To test if there were any other inhibitors unaccounted for in the synthesized CBA media, 20 g/L glucose was added to media with 40 g/L synthesized CBA. This mixed media produced 5.1 g/L isobutanol. Media with either 20 g/L glucose or 40 g/L synthesized CBA, produced 4 g/L or 2 g/L isobutanol, respectively (Figure 4). The additive isobutanol titer from these two carbon sources individually (6 g/L) is approximately the titer observed from the mixed (glucose and CBA) media (5.1 g/L).

### Isobutanol production from hydrolysate

**Figure 5:**
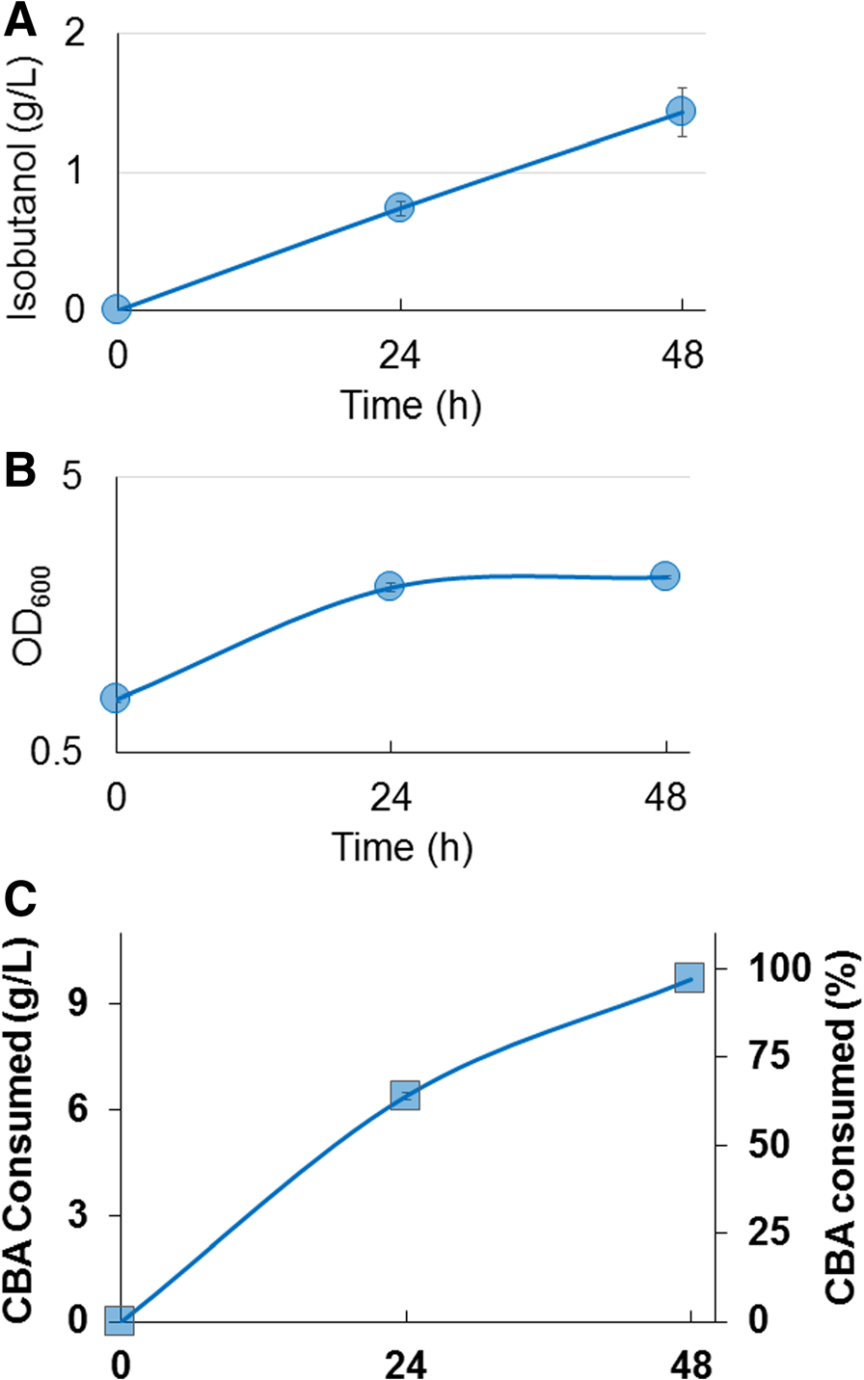
**Isobutanol Production from Hydrolysate. A**. Isobutanol titers from AL17(pAL603) grown in hydrolysate**. B.** Growth of AL17(pAL603) on hydrolysate during isobutanol production. **C.** Cellobionic acid consumed by AL17(pAL603) during isobutanol production. Where n = 3 biological replicates, and error bars represent standard deviation.

To demonstrate the feasibility of isobutanol production from CBA produced by fungal conversion of cellulose, a scenario that more closely mimics current multi-step biochemical biomass conversion methods, the engineered *E. coli* strain was grown in crude hydrolysate (Figure 5). The hydrolysate consisted of Avicel (crystalline cellulose) degraded by *N. crassa* into CBA (see Materials and Methods), after which *N. crassa* was removed from the media. The *E. coli* isobutanol producing strain was grown to OD_600_ ~ 0.6, washed, and then incubated with hydrolysate containing 9.7 g/L CBA. The *E. coli* cells were able to grow in the hydrolysate, which had been optimized for fungal growth and Avicel degradation (Figure 5B). Isobutanol was successfully produced at 36% of the theoretical maximum yield in 48 hours with a titer of 1.4 g/L, and a productivity of 0.03 g/L/h (Figure 5A).

## Discussion

Lignocellulosics are an abundant renewable feedstock with the potential to be converted into biofuels. However, there are several challenges that prevent straightforward industry-scale conversion of lignocellulosics into biofuels. One of these challenges is overcoming the recalcitrance of lignocellulosics to achieve efficient degradation. Another challenge is knowing the identity and relative ratio of minor degradation products such as CBA in lignocellulosic hydrolysate, since product content and ratios will vary depending on the source of lignocellulosic material and with the type of cellulase mixture utilized to degrade the lignocellulosic material. A promising key towards efficient lignocellulosic degradation is the recent understanding of LPMOs [11,12], which in turn presents a new challenge since the aldonic sugar products of these enzymes are compounds rarely utilized or studied in fermentation [13]. Therefore, this study aimed to understand the how CBA may be used as a carbon source for candidate biofuel production by the model organism *E. coli*, and so to make use of more total available lignocellulosic degradation products.

Cellobiose is similar in structure to CBA, and while cellobiose is naturally imported into *E. coli* by LacY [24], it would require activation of cryptic genes in order to metabolize cellobiose [25]. Thus, since typical growth conditions were used (that do not select for mutations that allow expression of cryptic genes), and since the *E. coli* strain used had a disrupted *lac* operon to enable blue-white colony screening, it seemed likely that, like cellobiose, CBA would not be naturally imported or metabolized by *E. coli*. Surprisingly, our *E. coli* strain AL17 (modified to optimize isobutanol production [19]) was able to grow using CBA as the sole carbon source (Figure 2). Furthermore, other *E. coli* strains such as MG1655, BL21(DE3) and XL-1 Blue also have the capacity to use CBA as a sole carbon source (Figure 2), suggesting that CBA is a natural metabolic substrate for *E. coli*. To identify the gene(s) required for growth on CBA, five genes were chosen for deletion from the *E. coli* genome. Though many of these genes were previously identified as cryptic [22,26,27], they were not excluded from consideration because they had been shown, upon activation, to allow growth on substrates similar in size and/or structure to that of CBA. We determined that AscB is at least primarily responsible for CBA metabolism since deletion and complementation of *ascB* abolished and restored growth on CBA, respectively (Figure 2). A deletion in *ascG*, the regulator of the *asc* operon, was previously thought to be the only means for activation of *ascB* expression [22,26]. However, sequencing revealed no mutations in any *asc* genes in *E. coli* strain AL17. Therefore, this study demonstrates that while *ascB* may be cryptic for expression in the presence of cellobiose, salicin and arbutin [24], it is not cryptic in the presence of CBA, and has a significant role in CBA *E. coli* metabolism. The mechanism of CBA entry into the cell is still unknown. However, CBA is a large charged molecule and most likely enters the cell through a specific (unidentified) transporter.

The *E. coli ascB* deletion strain provided a convenient tool to screen for other enzymes able to catabolize and allow growth on CBA. Previously, a beta-glucosidase (BglC) from *Thermobifida fusca* [28] was expressed in *E. coli* to allow its growth and isobutanol production using cellobiose [29]. When expressed in the AL17∆*ascB* strain, BglC was not able to rescue the growth defect on CBA. This demonstrates the specificity of this enzyme towards cellobiose. Alternatively, when the *N. crassa* CBAP was expressed extracellularly in *E. coli* [29,30]; it complemented for *E.coli* growth on CBA as a sole carbon source (Figure 2). It is unclear why AL17∆*ascB*(CBAP) had a long lag time in both glucose and CBA, but it may be attributed to CBAP extracellular expression. The ability of CBAP to function in *E. coli* demonstrates that this fungal enzyme was folded, exported, and functioned properly using *E. coli* machinery to mediate these events.

The isobutanol titer from 10.4 g/L commercial CBA was 2.7 g/L within 24 hours while that from 10 g/L glucose was 2.3 g/L, suggesting that commercial CBA is converted into isobutanol at comparable rates and efficiency as glucose. In contrast, the synthesized CBA (16.9 g/L) produced 2.4 g/L isobutanol while 3.6 g/L isobutanol was produced from 16 g/L glucose. Isobutanol titers were 30% less from synthesized CBA than from glucose. This indicated that a component of the mixture used to synthesize CBA may be inhibiting isobutanol production. Thus, each of the chemical components required for CBA synthesis were tested individually for inhibition of isobutanol production from glucose or gluconate, but no inhibitory affects were observed for any component (Figure 4). It is possible that one of the two enzymes used for synthesis of CBA may be inhibitory towards isobutanol production. However, production from mixed media containing both glucose and synthesized CBA resulted in 5.1 g/L isobutanol, a titer that was approximately the sum of the isobutanol titers from each carbon source individually (Figure 4). This suggests that the presence of these enzymes is unlikely the cause of low isobutanol titers from synthesized CBA. Additionally, it is unlikely that these enzymes would still be active after an over 24 hour incubation for CBA synthesis, and the subsequent change in conditions (pH and salts) when the CBA was combined with production media. Alternatively, it is possible that the two sources of CBA have different ratios of the free acid form versus the lactone form of CBA, which may affect CBA transport and metabolism in the cell. Identifying the specific difference(s) between commercial and synthesized CBA would contribute toward the understanding of CBA metabolism in living organisms, and may lead to more efficient methods for CBA synthesis, but is beyond the scope of this study.

All of the carbon in all media tested was consumed within 24 hours, suggesting that metabolism of glucose vs (either type of) CBA does not occur at significantly different rates. If CBA is degraded within the cell to G6P and gluconate as hypothesized, there is the possibility of carbon catabolite repression within the cell, wherein the presence of a preferred carbon source represses the metabolism of another carbon source, causing a delay in use of the total carbon in the culture media. This possibility was tested by mimicking the first step of CBA degradation by providing a 1:1 mix of glucose and gluconate, as substrate for isobutanol production (Figure 3). Isobutanol titers from this dual carbon media were similar to those of cultures with either glucose or gluconate alone, suggesting that carbon catabolite repression was not occurring during the time points analyzed in this study. This observation is in agreement with a similar finding by Fan et al. where glucose and gluconate are simultaneously utilized in an *E. coli* KO11 strain [15].

Redox balance has been crucial for high isobutanol titer and yield [31], especially in anaerobic conditions, although we used semi-aerobic conditions where redox balance has a decreased affect. For isobutanol production, 2 molecules of NADH are used for every molecule of isobutanol produced. In the case of synthesized CBA, which only achieved 36% of the theoretical maximum, it is possible that once CBA is broken down in the cell the 2 NADH are not regenerated, and so maximal isobutanol production is not achieved. Gluconate may be metabolized through either the Enter-Doudroff (ED) pathway or the pentose phosphate pathway [32-34]. Metabolism through the ED pathway would likely convert gluconate into pyruvate, the building block towards isobutanol synthesis. In contrast, gluconate metabolism through the pentose phosphate pathway would not produce the isobutanol precursor pyruvate. Carbon catabolite repression and co-factor regeneration are often reasons for low isobutanol yield. However, the high yield (65% of the theoretical maximum) of isobutanol from commercial CBA demonstrated in this study shows that these are not the challenges faced when using CBA as a substrate for target chemical production. The high yield also shows that this aldonic sugar would be a good lignocellulosic substrate for isobutanol production in a traditional multi-step (multi-organism) system. The discovery and partial understanding of CBA degradation by *E. coli* may contribute to optimization of future lignocellulosics-based biochemical production systems that contain CBA as an intermediate. Further studies identifying the difference between synthesized and commercial CBA will help achieve higher titer and yield from synthesized CBA.

We have also demonstrated that a target chemical can be produced from Avicel degraded into CBA by the fungus *N. crassa* (Figure 5). From this hydrolysate, 9.7 g/L CBA was converted to 1.4 g/L isobutanol, representing 36% of the theoretical maximum, the same yield as from defined production media with synthesized CBA. *E. coli* was able to grow in media that previously supported the growth of a fungus, and shows that metabolites produced by the fungus are not inhibitory towards *E. coli* growth. This production titer and yield can undoubtedly be improved, at least in part by understanding the differences of isobutanol production using CBA created by *in vitro* biological synthesis, *in vivo* by *N. crassa*, and chemical synthesis methods (Figure 5).

Previous studies of CBA have shown that although it is less inhibitory towards cellulases, its degradation by beta-glucosidases (an enzyme commonly used in lignocellulosic degradation) is slower than cellobiose by ten-fold and the gluconate product causes feedback inhibition [12]. However, natural CBA consumption by *E. coli* negates both of these concerns. The ability of *E. coli* to grow and produce isobutanol from the same media that supported growth of *N. crassa* shows that this engineered *E. coli* strain has potential application in a two-step (two-strain) biochemical production platform using cellulosic substrate. Future improvements to increase isobutanol titers and optimize media for growth of both organisms will further increase the industrial relevance of this system.

## Conclusions

In this study, it was identified that *E. coli* can naturally metabolize CBA, an important substrate in the process of lignocellulosic conversion into biofuels. Once thought to be a cryptic gene, *ascB,* was demonstrated as the gene primarily responsible for CBA metabolism in *E coli*. Furthermore, an *E. coli* strain optimized to produce isobutanol was successfully able to convert CBA into the biofuel candidate, isobutanol. To demonstrate industrial scale feasibility, *E. coli* was able to utilize CBA from fungus-treated hydrolysate and convert it into isobutanol. Though there is room for improvement in titer and yield for this system, this study demonstrates that the increasingly important aldonic acid CBA can be converted into a valuable biochemical.

## Materials and methods

### Reagents

All enzymes were purchased from New England Biolabs (Ipswich, MA). All synthetic oligonucleotides were ordered from Integrated DNA Technologies (Coralville, IA) or Eurofins Genomics (Huntsville, AL). DNA sequencing services were provided by Davis Sequencing (Davis, CA). All chemicals for gas chromatography (GC) standards were purchased from Sigma Aldrich (St. Louis, MO). Commercial cellobionic acid was purchased from MP Biomedicals (Santa Ana, CA).

### Plasmid and strain construction

All strains and plasmids used in this work are described in Table 1. All primers used are listed in Table 2. pAL952 was made by SLIC [35,36]. The vector backbone was amplified from pET-Duet (Merc Millipore (Billerica, MA)) using primers HY25/HY26 and the insert was amplified from genomic *E. coli* DNA by primers SD127/SD128. pAL959 was made via SLIC where the backbone was amplified from pZE12-luc [37] using primers SD11/SD12. The *ascB* gene insert was amplified from *E. coli* genomic DNA using primers SD136/SD137. pAL982 was made by digesting pAL959 and pAL450 with AvrII and SacI. The 2.7 and 2.3 KB products respectively were ligated together to form pAL982. pAL590 was made by SLIC where the vector was amplified from pSA69 using primers SD61/SD62 and the insert was amplified from pAL450 using primers SD59/SD60. pAL856 was made by SLIC where the vector backbone was amplified from pAL451 using primers YT571/YT572, and the insert was amplified from pAL590 using primers YT569/YT570. pAL869 was constructed via SLIC by amplifying the backbone from pAL856 using primers SD77/SD78 and the CBA phosphorylase gene insert was amplified from *N. crassa* genomic DNA using SD81/SD82. All plasmids were verified by restriction digest and sequencing. All genes were disrupted by the Wanner method [38].

**Table 2:** **Oligonucleotides used in this study**

**Primer Name**	**Primer Sequence**
SD11	ATGGTACCTTTCTCCTCTTTAATG
SD12	TAATCTAGAGGCATCAAATAAAAC
SD59	GCTTCCCAACCTTACCAGAGCTCGAGTCCCTATCAGTGATAG
SD60	CTCGAGGTGAAGACGAAAGGGCCACAACAGATAAAACGAAAGGCCCAGTC
SD61	CTCTGGTAAGGTTGGGAAGC
SD62	GGCCCTTTCGTCTTCACCTCGAG
SD77	TAAGGATCCTCTAGAGGCATCAAATAAAACG
SD78	ATTTTCCTGGTACGCGTAGCGGCCGCACTACCCTTAGTTTTC
SD81	AGTGCGGCCGCTACGCGTACCAGGAAAATGCCGACTCTGGTC
SD82	AGTGCGGCCGCTACGCGTACCAGGAAAATGCCGACTCTGGTC
SD127	AGCCATCACCATCATCACCACAGCTCAGTATTTCCAGAAAGTTTTTTATG
SD128	GTTATTGCTCAGCGGTGGCAGCAGTTACTGGCAATCACTTTTTTATAC
SD136	CATTAAAGAGGAGAAAGGTACCATGTCAGTATTTCCAGAAAGTTT
SD137	GTTTTATTTGATGCCTCTAGATTACCTACTCTAAATCTTCCCCATTACTGGCA
HY25	CTGCTGCCACCGCTGAGCAATAAC
HY26	GCTGTGGTGATGATGGTGATGGCT
YT569	CACCAGTGAAATCAAAGCCAAACTGC
YT570	TTTATTTGATGCCTCTAGAGGATCCTTATTCTTGACCGAAAATACCGCCATTAC
YT571	ATCCTCTAGAGGCATCAAATAAAACGAAAGGC
YT572	CAGCAGTTTGGCTTTGATTTCACTGG

### Cellobionic acid synthesis

Synthesized cellobionic acid was made by enzymatic conversion of 50 g/L cellobiose using 1 U/mL cellobiose dehydrogenase (CDH) (purified from *Pichia pastoris* [39]) 1 U/mL laccase from *Pleurotus ostreatus* (Sigma Aldrich (Saint Louis, MO)), 0.5 mM 2,2′-azino-bis(3-ethylbenzothiazoline-6-sulphonic acid (ABTS), and 30 mM sodium citrate mixed together to a total volume of 5 mL in a 125 mL baffled flask. This solution was kept at 30°C, 250 rpm for 24 hours. Complete conversion of cellobiose to CBA was confirmed with LC-MS (UC Davis Mass Spec facility) [17].

### Culture conditions

All strains were cultured with appropriate antibiotics (ampicillin (100 μg/mL), chloramphenicol (40 μg/mL), kanamycin (50 μg/mL), tetracycline (10 μg/mL)). Minimal media was used for growth assays. It contained: 1X M9 salts (33.9 g/L Na_2_HPO_4_, 15 g/L KH_2_PO_4_, 2.5 g/L NaCl, 5 g/L NH_4_Cl), 2 g/L of the appropriate sugars, 1 mM MgSO_4_, and 0.1 mM CaCl_2_. Production media was used for isobutanol production assays. It contained: 1X M9 salts, 5 g/L yeast extract, 1 mM MgSO_4_, 0.1 mM CaCl_2_, and appropriate sugars. All cultures were incubated at 37°C with constant (250 rpm) shaking unless otherwise specified.

### Growth assay

1 mL of overnight culture (at an OD_600_ of approximately 1) was harvested by centrifugation at 1,500 x *g* for 3 minutes, resuspended in 1 mL of M9 media without a carbon source, centrifuged then resuspended in 100 μL of M9 media without a carbon source. Concentrated cells were inoculated to an OD_600_ ~ 0.1 into M9 media containing 2 g/L of either glucose or cellobionic acid. Cultures were incubated at 37°C, 250 rpm. OD_600_ was measured at regular intervals until stationary phase was achieved by all cultures. All experiments were performed with three biological replicates.

### Isobutanol production

Production media (5 mL) with appropriate antibiotics was inoculated with 1% of overnight culture. The cultures were grown at 37°C with shaking (250 rpm) until any strain reached OD_600_ ~ 0.4 (approximately 4 hours). 0.1 mM IPTG was then added and the strains were grown at 30°C for 48 hours. Samples were harvested by centrifugation at 1,500 x *g* for 3 minutes, and the supernatant was used to measure isobutanol and substrate concentration by GC and high performance liquid chromatography (HPLC) analysis, respectively. All experiments were performed with three biological replicates.

### Preparation of hydrolysate

*N. crassa* F5 ∆*cre* ∆*ace* ∆*nvdB*, OD_420_ of 0.1 was inoculated into Vogel’s media supplemented with 20 g/L Avicel and 0.6 g/L glucose, and grown for 96 hours at room temperature, 200 rpm [17]. After the 96 hour incubation, the cellobiose to cellobionate conversion was begun by the addition of 0.8 U/mL laccase and 0.1 mM ABTS [17]. The conversion was allowed to continue for 24 hours, and the hydrolysate was then filtered through a 0.22 μm filter to remove cells.

### Isobutanol production from hydrolysate

Production media (10 mL) with 10 g/L glucose was inoculated with 1% overnight culture of AL17 harboring pAL603 grown at 37°C, 250 rpm until an OD_600_ of 0.4 was reached, upon which 0.1 mM IPTG was added and the cells were shifted to 30°C, 250 rpm for 3 hours. Thereafter the cells were harvested by centrifugation at 1,500 × *g* for 5 minutes, washed in M9 media without a carbon source and resuspended in 10 mL of hydrolysate (pH7) with 0.1 mM IPTG. The cells were then incubated at 30°C, 250 rpm for the duration of the experiment.

### HPLC analysis

Glucose consumption was measured using a Shimadzu 20A HPLC (Columbia, MD) equipped with a differential refractive detector (RID) 10A and a Bio-Rad (Hercules, CA, USA) Aminex fast acid analysis column. 5 mM H_2_SO_4_ served as the mobile phase at a flow rate of 0.6 mL/min at 65°C for 12.5 minutes. Cellobionic acid consumption was measured using an ICSep ICE-ION300 from Transgenomics (Omaha, NE). 5 mM H_2_SO_4_ served as the mobile phase at a flow rate of 0.5 mL/min at 80°C for 15 minutes.

### GC analysis

Isobutanol production was analyzed by GC (Shimadzu GC-2010) equipped with a flame ionization detector (FID) and the FFAP capillary column (60 m length, 0.32-mm diameter, 1-μm film thickness) from Agilent Technologies (Santa Clara, CA, USA). GC oven temperature was initially held at 40°C for 3 minutes, then increased at a rate of 45°C min^−1^ to 235°C and held for 4 minutes. Injector temperature was held at 225°C and FID detector temperature was held at 330°C. Injection volume was 0.5 μL, injected at a 15:1 split ratio. Helium was used as the carrier gas. 1-Pentanol was used as an internal standard.

### Yield calculations

Yield was calculated by using the following stoichiometric ratios.

Cellobionic acid

C_12_H_22_O_12_ → 1.92 C_4_H_10_O + 4.33 CO_2_ + 1.42 H_2_O

Stoichiometric conversion gives the following ratio: 0.4 g isobutanol/g CBA

Gluconate

C_6_H_12_O_7_ → 0.92 C_4_H_10_O + 2.33 CO_2_ + 1.42 H_2_O

Stoichiometric conversion gives the following ratio: 0.35 g isobutanol/g gluconate

Glucose

C_6_H_12_O_6_ → C_4_H_10_O + 2 CO_2_ + H_2_O

Stoichiometric conversion gives the following ratio: 0.41 g isobutanol/g glucose

## Abbreviations

CBA: Cellobionic Acid
CBAP: Cellobionic acid phosphorylase

## References

[1] Shafiee S, Topal E (2009). When will fossil fuel reserves be diminished?. Energ Pol.

[2] Orts WJ, Holtman KM, Seiber JN (2008). Agricultural chemistry and bioenergy. J Agric Food Chem.

[3] Stamm P, Verma V, Ramamoorthy R, Kumar PP (2012). Manipulation of plant architecture to enhance lignocellulosic biomass. AoB Plants.

[4] Luo D, Hu Z, Choi DG, Thomas VM, Realff MJ, Chance RR (2010). Life cycle energy and greenhouse gas emissions for an ethanol production process based on blue-green algae. Environ Sci Technol.

[5] Geddes CC, Nieves IU, Ingram LO (2011). Advances in ethanol production. Curr Opin Biotechnol.

[6] Hahn-Hagerdal B, Galbe M, Gorwa-Grauslund MF, Liden G, Zacchi G (2006). Bio-ethanol–the fuel of tomorrow from the residues of today. Trends Biotechnol.

[7] Olson DG, McBride JE, Shaw AJ, Lynd LR (2012). Recent progress in consolidated bioprocessing. Curr Opin Biotechnol.

[8] Blanch HW (2012). Bioprocessing for biofuels. Curr Opin Biotechnol.

[9] Yamada R, Hasunuma T, Kondo A (2013). Endowing non-cellulolytic microorganisms with cellulolytic activity aiming for consolidated bioprocessing. Biotechnol Adv.

[10] Lynd LR, Van Zyl WH, McBride JE, Laser M (2005). Consolidated bioprocessing of cellulosic biomass: an update. Curr Opin Biotechnol.

[11] Isaksen T, Westereng B, Aachmann FL, Agger JW, Kracher D, Kittl R (2014). A C4-oxidizing lytic polysaccharide monooxygenase cleaving both cellulose and cello-oligosaccharides. J Biol Chem.

[12] Horn SJ, Vaaje-Kolstad G, Westereng B, Eijsink VG (2012). Novel enzymes for the degradation of cellulose. Biotechnol Biofuels.

[13] Cannella D, Hsieh CW, Felby C, Jorgensen H (2012). Production and effect of aldonic acids during enzymatic hydrolysis of lignocellulose at high dry matter content. Biotechnol Biofuels.

[14] Igarashi K, Samejima M, Eriksson KE (1998). Cellobiose dehydrogenase enhances *Phanerochaete chrysosporium* cellobiohydrolase I activity by relieving product inhibition. Eur J Biochem.

[15] Fan Z, Wu W, Hildebrand A, Kasuga T, Zhang R, Xiong X (2012). A novel biochemical route for fuels and chemicals production from cellulosic biomass. Plos One.

[16] Nihira T, Saito Y, Nishimoto M, Kitaoka M, Igarashi K, Ohtsubo K (2013). Discovery of cellobionic acid phosphorylase in cellulolytic bacteria and fungi. FEBS Lett.

[17] Hildebrand A, Szewczyk E, Lin H, Kasuga T, Fan Z (2014). Engineering *Neurospora crassa* for improved cellobiose and cellobionate production. Appl Environ Microbiol.

[18] Rude MA, Schirmer A (2009). New microbial fuels: a biotech perspective. Curr Opin Microbiol.

[19] Atsumi S, Hanai T, Liao JC (2008). Non-fermentative pathways for synthesis of branched-chain higher alcohols as biofuels. Nature.

[20] Rabinovitch-Deere CA, Oliver JW, Rodriguez GM, Atsumi S (2013). Synthetic biology and metabolic engineering approaches to produce biofuels. Chem Rev.

[21] Nozzi NE, Desai SH, Case AE, Atsumi S (2014). Metabolic engineering for higher alcohol production. Metab Eng.

[22] Hall BG, Xu L (1992). Nucleotide sequence, function, activation, and evolution of the cryptic asc operon of *Escherichia coli* K12. Mol Biol Evol.

[23] Kitagawa M, Ara T, Arifuzzaman M, Ioka-Nakamichi T, Inamoto E, Toyonaga H (2005). Complete set of ORF clones of *Escherichia coli* ASKA library (a complete set of *E. coli* K-12 ORF archive): unique resources for biological research. DNA Res.

[24] Sekar R, Shin HD, Chen R (2012). Engineering *Escherichia coli* cells for cellobiose assimilation through a phosphorolytic mechanism. Appl Environ Microbiol.

[25] Parker LL, Hall BG (1990). Mechanisms of activation of the cryptic cel operon of *Escherichia coli* K12. Genetics.

[26] Ishida Y, Kori A, Ishihama A (2009). Participation of regulator AscG of the beta-glucoside utilization operon in regulation of the propionate catabolism operon. J Bacteriol.

[27] Keyhani NO, Roseman S (1997). Wild-type Escherichia coli grows on the chitin disaccharide, N, N’-diacetylchitobiose, by expressing the cel operon. Proc Natl Acad Sci U S A.

[28] Spiridonov NA, Wilson DB (2001). Cloning and biochemical characterization of BglC, a beta-glucosidase from the cellulolytic actinomycete *Thermobifida fusca*. Curr Microbiol.

[29] Desai SH, Rabinovitch-Deere CA, Tashiro Y, Atsumi S (2014). Isobutanol production from cellobiose in *Escherichia coli*. Appl Microbiol Biotechnol.

[30] Qian ZG, Xia XX, Choi JH, Lee SY (2008). Proteome-based identification of fusion partner for high-level extracellular production of recombinant proteins in *Escherichia coli*. Biotechnol Bioeng.

[31] Bastian S, Liu X, Meyerowitz JT, Snow CD, Chen MM, Arnold FH (2011). Engineered ketol-acid reductoisomerase and alcohol dehydrogenase enable anaerobic 2-methylpropan-1-ol production at theoretical yield in *Escherichia coli*. Metab Eng.

[32] Conway T (1992). The Entner-Doudoroff pathway: history, physiology and molecular biology. FEMS Microbiol Rev.

[33] Eisenberg RC, Dobrogosz WJ (1967). Gluconate metabolism in *Escherichia coli*. J Bacteriol.

[34] Hanke T, Noh K, Noack S, Polen T, Bringer S, Sahm H (2013). Combined fluxomics and transcriptomics analysis of glucose catabolism via a partially cyclic pentose phosphate pathway in *Gluconobacter oxydans* 621H. Appl Environ Microbiol.

[35] Li MZ, Elledge SJ (2007). Harnessing homologous recombination *in vitro* to generate recombinant DNA via SLIC. Nat Methods.

[36] Machado HB, Dekishima Y, Luo H, Lan EI, Liao JC (2012). A selection platform for carbon chain elongation using the CoA-dependent pathway to produce linear higher alcohols. Metab Eng.

[37] Lutz R, Bujard H (1997). Independent and tight regulation of transcriptional units in *Escherichia coli* via the LacR/O, the TetR/O and AraC/I1-I2 regulatory elements. Nucleic Acids Res.

[38] Datsenko KA, Wanner BL (2000). One-step inactivation of chromosomal genes in *Escherichia coli* K-12 using PCR products. Proc Natl Acad Sci U S A.

[39] Zhang R, Fan Z, Kasuga T (2011). Expression of cellobiose dehydrogenase from *Neurospora crassa* in *Pichia pastoris* and its purification and characterization. Protein Expr Purif.

